# Dicumarol inhibits PDK1 and targets multiple malignant behaviors of ovarian cancer cells

**DOI:** 10.1371/journal.pone.0179672

**Published:** 2017-06-15

**Authors:** Wenjia Zhang, Jing Su, Huadan Xu, Shanshan Yu, Yanan Liu, Yong Zhang, Liankun Sun, Ying Yue, Xiaoli Zhou

**Affiliations:** 1 Department of Gynecological Oncology, The First Hospital of Jilin University, Changchun, China; 2 College of Basic Medical Sciences, Jilin University, Changchun, China; 3 State Key Laboratory of Microbial Metabolism, School of Life Sciences and Biotechnology, Shanghai Jiao Tong University, Shanghai, China; University of South Alabama Mitchell Cancer Institute, UNITED STATES

## Abstract

Pyruvate dehydrogenase kinase 1 (PDK1) is overexpressed in ovarian cancer and thus is a promising anticancer therapeutic target. Our previous work suggests that coumarin compounds are potential inhibitors of PDKs. In this study, we used the ovarian cancer cell line SKOV3 as the model system and examined whether dicumarol (DIC), a coumarin compound, could inhibit ovarian cancer through targeting PDK1. We showed that DIC potently inhibited the kinase activity of PDK1, shifted the glucose metabolism from aerobic glycolysis to oxidative phosphorylation, generated a higher level of reactive oxygen species (ROS), attenuated the mitochondrial membrane potential (MMP), induced apoptosis, and reduced cell viability *in vitro*. The same phenotypes induced by DIC also were translated *in vivo*, leading to significant suppression of xenograft growth. This study not only identifies a novel inhibitor for PDK1, but it also reveals novel anticancer mechanisms of DIC and provides a promising anticancer therapy that targets the Warburg effect.

## Introduction

Ovarian cancer cells, similar to other solid tumor cells and in contrast to normal cells, heavily rely on aerobic glycolysis for energy production, a phenomenon known as the Warburg effect, which is closely associated with malignant behaviors of tumors, including metastatic spread and the development of resistance to chemo- or radiotherapy [1]. Multiple mechanisms are responsible for the Warburg effect, mostly the dysregulation of enzymes involved in glucose metabolism. Pyruvate dehydrogenase kinase 1 (PDK1) is the key enzyme that negatively regulates the activity of the pyruvate dehydrogenase complex (PDC) through phosphorylation [2, 3]. Since the inactivation of the PDC is tightly associated with the Warburg effect, several recent studies have demonstrated that PDK1 plays a major role in the metabolism of cancer cells [4, 5]; not only are the expression and activity of PDK1 strongly regulated by oncogenes [6], but PDK1 is universally overexpressed in a variety of cancer cells, such as ovarian cancer, multiple myeloma, and breast cancer [1, 7–10]. Therefore, targeting PDK1 provides an attractive therapeutic strategy for multiple cancers.

Several small-molecule inhibitors have been developed to regulate PDK1 activity. For example, dichloroacetate (DCA) targets the pyruvate-binding site [11, 12]; AZD7545 targets the lipoamide-binding pocket [13, 14]; and radicicol [15], JX06 [16], and VER-246608 [17] target the nucleotide-binding pocket of PDK1. However, no studies have investigated the anticancer effects of AZD7545 or VER-246608. Although radicicol presents some antitumor activity [15], the underlying mechanisms remain unknown. DCA and JX06 have been demonstrated to target cancer development through modulating glucose metabolism, increasing reactive oxygen species (ROS), decreasing the mitochondrial membrane potential (MMP), and initiating apoptosis [16, 18]

So far, only DCA has entered clinical trials, but the high effective dosage and side effects have limited its application [19, 20]. Therefore, it is imperative to develop potent PDK1 inhibitors with a high safety profile and minimal side effects as well as to examine their antitumor activities to assess their therapeutic potential in cancer treatment.

Coumarins are a family of compounds widely distributed in plants, bacteria, and fungi; they present various pharmacological properties, including antibacterial [21], antimutagenic [22], anti-inflammatory [23], anticoagulant [24–26], antithrombotic [27, 28], and anticancer activities [29]. In cancers, coumarins target a number of pathways or signaling molecules, including cell cycle progression, angiogenesis, heat shock protein 90, telomerase, mitosis, carbonic anhydrase, monocarboxylate transporters, aromatase, and sulfatase [30]. Dicumarol (DIC) is a coumarin that disrupts the vitamin K cycle and thus is widely used as an anticoagulant [31]. Recent work also has revealed the anticancer effects of DIC [30–36], which in some studies are attributed to the inhibition of NAD(P)H:quinone oxidoreductase 1 (NQO1) [32–35], and in others, to the suppression of chemoresistance by targeting pregnancy-specific beta-1-glycoprotein [36]. Moreover, the anticancer effects of DIC have been linked to the induction of apoptosis in cancer cells by blocking stress-activated protein kinase/c-Jun N-terminal kinase and nuclear factor-κB pathways [37, 38].

Generating a high level of ROS is considered a pivotal antitumor mechanism of DIC, leading to the inhibition of NQO1 [30–33]. In ovarian cancer cells, however, it is not known how DIC elevates the level of ROS. Furthermore, oxidative phosphorylation is a well-demonstrated source for ROS production. In our previous work, using structure-based virtual screening, we found that coumarin compounds might inhibit PDKs through binding to lipoamide-binding pockets, thus preventing PDK from binding to PDC. Therefore, targeting the Warburg effect and increasing the ROS level may be a novel mechanism for the anticancer activities of coumarins [39].

In the present study, we used the ovarian cancer cell lines SKOV3 and A2780 as the model system, assessed the effect of DIC on PDK1 activity, and investigated the anticancer activities of DIC both *in vitro* and *in vivo*. Our findings not only suggest a novel mechanism by which DIC may affect the Warburg effect but also provide evidence that DIC may be an effective therapeutic agent against cancer.

## Materials and methods

### Molecular docking

The CDOCKER module of Discovery Studio 3.5 (DS3.5, Accelrys, Inc., San Diego, CA, USA) was used for the molecular docking study, as described previously [39]. In brief, the crystal structure of PDK1 (PDB ID: 2Q8G, 1.9 Å) was obtained from the Protein Data Bank. The water molecules were removed and hydrogen atoms were added to the protein. The three-dimensional structure of DIC was obtained from the PubChem database. The CHARMM force field was used for the receptor and ligand. The binding-site sphere of PDK1 was defined as the region that comes within a 15-Å radius from the geometric centroid of the ligand AZD7545.

### Reagents and antibodies

DIC was purchased from Selleckchem (Houston, TX, USA) and was dissolved in 2% dimethyl sulfoxide (DMSO). The glycolysis inhibitor DCA, 3-(4, 5-dimethylthiazol-2-yl)-2, 5-diphenyltetrazolium bromide (MTT), and Hoechst 33342 were purchased from Sigma (Shanghai, China). Anti-caspase-3, anti-cleaved-caspase-3, anti-poly (ADP-ribose) polymerase (PARP), anti-cleaved-PARP, and anti-β-actin antibodies were purchased from Santa Cruz Biotechnology (Dallas, TX, USA). Anti-PDK1, anti-PDH, and anti-p-PDH antibodies were bought from Abcam Biotechnology (Cambridge, MA, USA). The terminal deoxynucleotidyl transferase-mediated dUTP nick-end labeling (TUNEL) kit was purchased from Roche (Basel, Switzerland).

### Cell culture

The human ovarian cancer cell lines SKOV3 and A2780 were purchased from the American Tissue Culture Collection (Rockville, MD, USA) and Shanghai Kenqiang Equipment Co., Ltd. (Shanghai, China), respectively. Both cell lines were cultured in RPMI-1640 medium containing 10% fetal bovine serum and 1% antibiotics (Hyclone, Logan, UT, USA). They grew in a humidified cell culture incubator containing 5% CO_2_ and 95% air at 37°C.

### Enzyme-linked immunosorbent assay (ELISA)-based PDK1 activity assay

Since PDK1 specifically phosphorylates PDH E1 alpha protein (PDHA1) at serine 232 [40], we detected the effect of DIC or DCA on PDK1 enzymatic activity using the phospho-S232 PDHA1 Profiling ELISA Kit (Cat. No. ab115343, Abcam), according to the manufacturer’s instructions. Briefly, SKOV3 cells were lysed in ice-cold radioimmunoprecipitation assay (RIPA) buffer containing 1% phenylmethane sulfonyl fluoride (PMSF) and 1% β-mercaptoethanol, and the lysate was collected by centrifugation at 12,000 × *g* and 4°C for 10 min. After measuring the protein concentrations of the lysate, 12.5 μg of total protein was added into the 96-well microplate coated with a capture antibody for PDHA1. After three washes using the wash buffer provided with the kit, a preheated mixture of active PDK1 (2 μg/well; Abcam) and the phosphorylation buffer, with or without DIC or DCA, was added into each well and incubated at 30°C for 10 min. After another three washes, phospho-S232 PDHA1 detector antibody was added and incubated at room temperature for 1 h. Then, the wells were washed and incubated with horseradish peroxidase (HRP)-labeled probe at room temperature for 1 h, followed by signal development using the HRP substrate (3,3',5,5'-tetramethylbenzidine) solution provided with the kit. The reaction was stopped by the addition of 100 μL of 1 M HCl, the signal in each well was recorded by reading the optical density at 450 nm, and the phospho-S232 PDHA1 concentration was calculated based on the standard curve.

### Cell viability assay

The *in vitro* cell viability was examined using the standard MTT assay, as described previously [41]. Briefly, SKOV3 or A2780 cells were seeded in 96-well plates at 8000 cells/well. The next day, increasing concentrations of DIC were added into each well, and the plate was incubated for 24 h. Then, 10 μL of 10 mg/mL MTT reagent (Sigma, Shanghai, China) in phosphate-buffered saline (PBS) was added into each well, and the plate was incubated for an additional 4 h. The formazan crystals were dissolved in 150 μL of DMSO, and after the plate was shaken for 5 min, the optical density at 570 nm was recorded by the ELISA reader.

### Western immunoblotting

Drug-treated cells were lysed in cold RIPA buffer containing 1% PMSF and 1% β-mercaptoethanol, and the lysate was collected by centrifuging at 12,000 × *g* for 10 min at 4°C. The total protein in each sample was quantified using the Bio-Rad protein reagent (Bio-Rad Laboratories, Hercules, CA, USA). Approximately 50 μg of total protein from each sample was denatured at 95°C for 10 min, separated by 12–15% sodium dodecyl sulfate—polyacrylamide gel electrophoresis, and transferred to Immune-Blot polyvinylidene fluoride membranes (Bio-Rad Laboratories). After blocking in 10% (w/v) nonfat milk in Tris-buffered saline for 1 h, the membranes were incubated with specific primary antibodies overnight at 4°C. Following incubation of the membranes with horseradish peroxidase-conjugated secondary antibodies for 2 h, the signals were detected using enhanced chemiluminescence reagents followed by Syngene Bio Imaging (Synoptics, Cambridge, UK). The band densities were measured using Syngene Bio Imaging tools, as described previously [41].

### Hoechst 33342 staining assay

We detected morphological alterations of apoptotic cells by staining the nuclear chromatin of SKOV3 cells with Hoechst 33343. In brief, SKOV3 cells were cultured in 24-well plates and treated as indicated for an additional 24 h. The cells were washed with cold PBS and fixed using 4% (w/v) paraformaldehyde for 15 min. The plates were then incubated with 1 μg/mL Hoechst 33343 (Santa Cruz) for 10 min and observed under a fluorescence microscope (IX-71, Olympus), as described previously [41].

### Apoptosis assay

SKOV3 or A2780 cells were seeded into 6-well plates and divided into six groups according to the treatments they received: control (unstained), control (stained), DMSO (2%), DCA (50 mM), DIC (100 μM), and DIC (200 μM). Following the 24-h treatment, apoptosis was measured by staining the cells with annexin V and propidium iodide (PI) using the FITC Annexin V Apoptosis Detection Kit from BD Pharmingen (Shanghai, China). The cells were analyzed on a C6 Flow Cytometer, and the signal was quantified using C6 Software and a Workstation Computer (BD AccuriTM), as described previously [16].

### Determination of glucose uptake and lactate production

SKOV3 or A2780 cells were treated with the indicated drugs for 24 h, washed with PBS, and cultured in RPMI-1640 culture medium to achieve a confluency of 70%. The culture medium was then collected, and the glucose and lactate concentrations were measured with a glucose assay kit and a lactate assay kit (Beyotime, Jiangsu, China), respectively. The data were normalized by the corresponding total protein amounts from each sample, as described previously [41].

### Oxygen consumption rate (OCR) and extracellular acidification rate (ECAR) analysis

A total of 8 × 10^4^ SKOV3 or A2780 cells were seeded into 96-well plates and incubated overnight to allow adherence. The following day, different concentrations of drugs were added into the indicated wells. Each treatment was repeated in three wells. The OCR and ECAR were measured using oxygen-sensitive (Mito-Xpress) and pH-sensitive (pH-Xtra) fluorescent probes (Luxcel Bioscience, Cork, Ireland), as described previously [41]

### ROS measurement

SKOV3 or A2780 cells were treated with 2% DMSO, 300 μM H_2_O_2_, 50 mM DCA, 100 μM DIC, or 200 μM DIC for 24 h, and then the ROS level in each sample was detected by cell staining with MitoSOX^™^ Red Mitochondrial Superoxide Indicator (Invitrogen, Carlsbad, CA, USA), according to the manufacturer’s instructions. Positive cells containing a high level of ROS were detected by flow cytometry, as described previously [16].

### MMP measurement

The MMP was determined by using JC-1 dye, contained within the Mitochondrial Membrane Potential Assay Kit (Beyotime). At 24 h after DIC treatment, the cells were incubated with 1 mL of 1× JC-1 for 30 min at 37°C in the dark, and the ratio of cells positive for red fluorescence (JC-1 polymer indicating intact MMP) to those positive for green fluorescence (monomeric form of JC-1, an indicator for loss of MMP) was determined by flow cytometry using BD Accuri C6, as described previously [42].

### Mouse experiments

Twenty-five female BALB/c-nu mice aged 5–6 weeks old and weighing approximately 15 g each were purchased from the Animal Experimental Center (Beijing, China). A total of 1 × 10^7^ SKOV3 cells were subcutaneously injected into the upper flank. After 10 days, when the tumor volume reached approximately 100 mm^3^, the nude mice were randomized into five groups (n = 5/group) and were given the following treatments intraperitoneally (i.p.) every other day, for a total of 12 days: control group, administered with 0.2 mL of 0.9% NaCl; vehicle group, administered with 1 mM NaOH [43]; DCA group, administered with 100 mg/kg DCA; DIC-30 group, administered with 30 mg/kg DIC; and DIC-50 group, administered with 50 mg/kg DIC. The body weights and tumor volumes of each mouse were monitored every other day until sacrifice (on day 12 after the initial treatment). All animal experiments were approved by the Animal Welfare and Ethics Group of the Laboratory Animal Science Department, Jilin University (Changchun, China).

### Immunohistochemistry

Mouse tissues were fixed in 4% (w/v) paraformaldehyde, dehydrated in graded ethanol, and embedded in paraffin. Samples were then cut into 3-μm sections using a Leica microtome. TUNEL and p-PDHA1 staining were carried out using the corresponding kits, according to the manufacturer’s instructions. Sections were analyzed using an inverted fluorescence microscope (Olympus, Japan).

### Statistical analysis

Data were expressed as the mean ± standard error (SE). Statistical significance between two groups was calculated using the Student’s *t*-test of SPSS12.0 software. * represents P < 0.05, ** represents P < 0.01, and *** represents P < 0.001. P < 0.05 was considered statistically significant. All experiments were repeated at least three times.

## Results

### DIC inhibits the kinase activity of PDK1 in vitro

**Fig 1:**
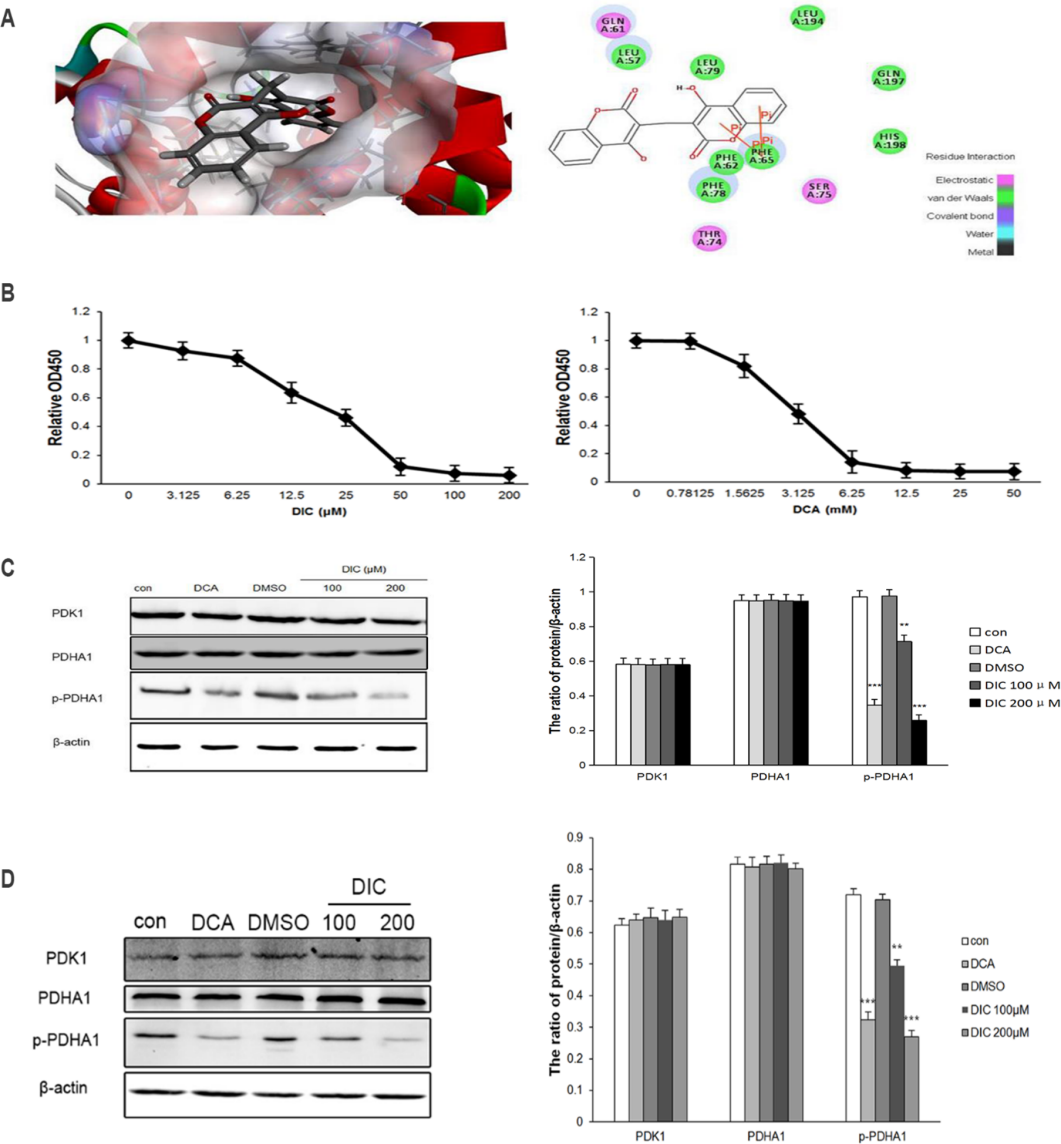
DIC inhibits PDK1 activity *in vitro*. **(A)** Molecular docking showing the potential interaction between DIC and PDK1. Left panel, schematic figure showing the surface of the lipoamide-binding pocket, with blue representing a positive charge, red representing a negative charge, and DIC shown in stick form; right panel, the residues from PDK1 that are predicted to interact with DIC and their mode of interactions. **(B)** In-well kinase assay measuring PDK1 enzymatic activity. Increasing concentrations of DIC (left panel) or DCA (right panel) were added to a 96-well plate. The enzymatic activity of PDK1 was measured by a microplate reader. **(C)** SKOV3 cells were treated as indicated. The levels of PDK1, PDHA1, and p-PDHA1 were measured using western immunoblotting. β-Actin was detected as the internal control. Representative western blot images are shown on the left, and the quantitation of PDK1, PDHA1, and p-PDHA1 protein levels relative to β-actin are shown on the right. **(D)** A2780 cells were treaed s indicated. The protein levels of PDK1, PDHA1, and p-PDHA1 were examined by western immunoblotting, with β-actin examined as the internal control. Representative western blot images are shown on the left, and the quantification of protein levels relative to β-actin are shown on the right. Data are presented as the mean ± SE from three independent experiments. *P < 0.05, **P < 0.01, ***P < 0.001, when compared to nontreated control cells (con).

To understand the potential interaction between DIC and PDK1, we performed molecular docking using the CDOCKER module of Discovery Studio 3.5. The CDOCKER interaction energy of the resulting PDK1-DIC complex, which represents the molecular complex binding affinity, was determined to be -33.65 kcal/mol. Molecular docking also revealed that intermolecular interactions formed between DIC and PDK1 (Fig 1A): the centroids of the benzene ring of the coumarin groups of DIC formed two pairs of pi-pi interactions with the centroids of the benzene ring of PHE65, which resides in the lipoamide-binding pocket of PDK1; DIC formed van der Waals interactions with residues LEU57, PHE62, PHE65, PHE78, LEU79, LEU194, GLN197, and HIS198, and electrostatic interactions with residues GLN61, THR74, and SER75 of PDK1. All these interactions suggested that DIC may bind to the lipoamide-binding pocket of PDK1 and thus inhibit its activity.

To test the validity of the predicted interaction between DIC and PDK1, we measured the kinase activity of PDK1 in response to DIC using an *in vitro* biochemical enzymatic assay, and DCA was applied as a positive control. As shown in Fig 1B, the PDK1 activity was inhibited by DIC in a dose-dependent manner. The enzymatic activity of PDK1 was reduced by approximately 94% when treated with 200 μM DIC. DIC also presented a much higher potency than the well-characterized PDK1 inhibitor DCA, with the half-maximal inhibitory concentration (IC_50_) for DIC being 19.42 ± 0.032 μM and that for DCA being 3.042 ± 0.087 mM (Fig 1B).

These findings prompted us to further explore whether DIC inhibits PDK1 enzymatic activity in cancer cells. In this study, we focused on the ovarian cancer cell lines SKOV3 and A2780. Inhibition of PDK1 activity is closely linked to the activation of PDC, as represented by reduced levels of phosphorylated PDHA1. Upon treatment of SKOV3 cells with DIC (100 μM or 200 μM), we examined the level of phosphorylated PDHA1 (p-PDHA1) at SER232, a site specifically and uniquely catalyzed by PDK1 [40]. The results showed that DIC decreased the p-PDHA1 level by 26% (100 μM DIC) and by 72% (200 μM DIC), with no statistical difference in the total PDHA1 level (Fig 1C). To understand whether the reduction of p-PDHA1 is due to a decrease in the level of PDK1 or the enzymatic activity of PDK1, we measured the level of PDK1 in response to DIC. As shown in Fig 1C, 100 μM or 200 μM DIC did not alter the protein level of PDK1, suggesting that DIC inhibits the kinase activity of PDK1 rather than its expression level in SKOV3 cells. Similarly, in A2780 cells, the p-PDHA1 level was decreased by 20.1% in 100 μM DIC-treated, and by 43.5% in 200 μM DIC-treated A2780 cells, while the levels of total PDHA1 and PDK1 were not significantly altered by DIC (Fig 1D). In SKOV3 cells, the following PDHA1 levels were observed for DCA-, DMSO-, 100 μM DIC-, and 200 μM DIC-treated cells: 0.2% lower than that of the control, 0.2% higher than that of the control, 0.1% lower than that of the control, and 0.3% lower than that of the control, respectively. In SKOV3 cells, the following PDK1 levels were observed for the same groups: 0.1% lower than that of the control, 0.1% lower than that of the control, 0.1% higher than that of the control, and 0.1% lower than that of the control, respectively (Fig 1C). In A2780 cells, the following PDHA1 levels were observed for the same groups: 0.8% lower than that of the control, 0.01% higher than that of the control, 0.4% higher than that of the control, and 1.5% lower than that of the control, respectively. In A2780 cells, the following PDK1 levels were observed for the same groups: 1.5%, 2%, 1.6%, and 2.4% higher than that of the control, respectively (Fig 1D).

### DIC alters glucose metabolism in ovarian cancer cells

**Fig 2:**
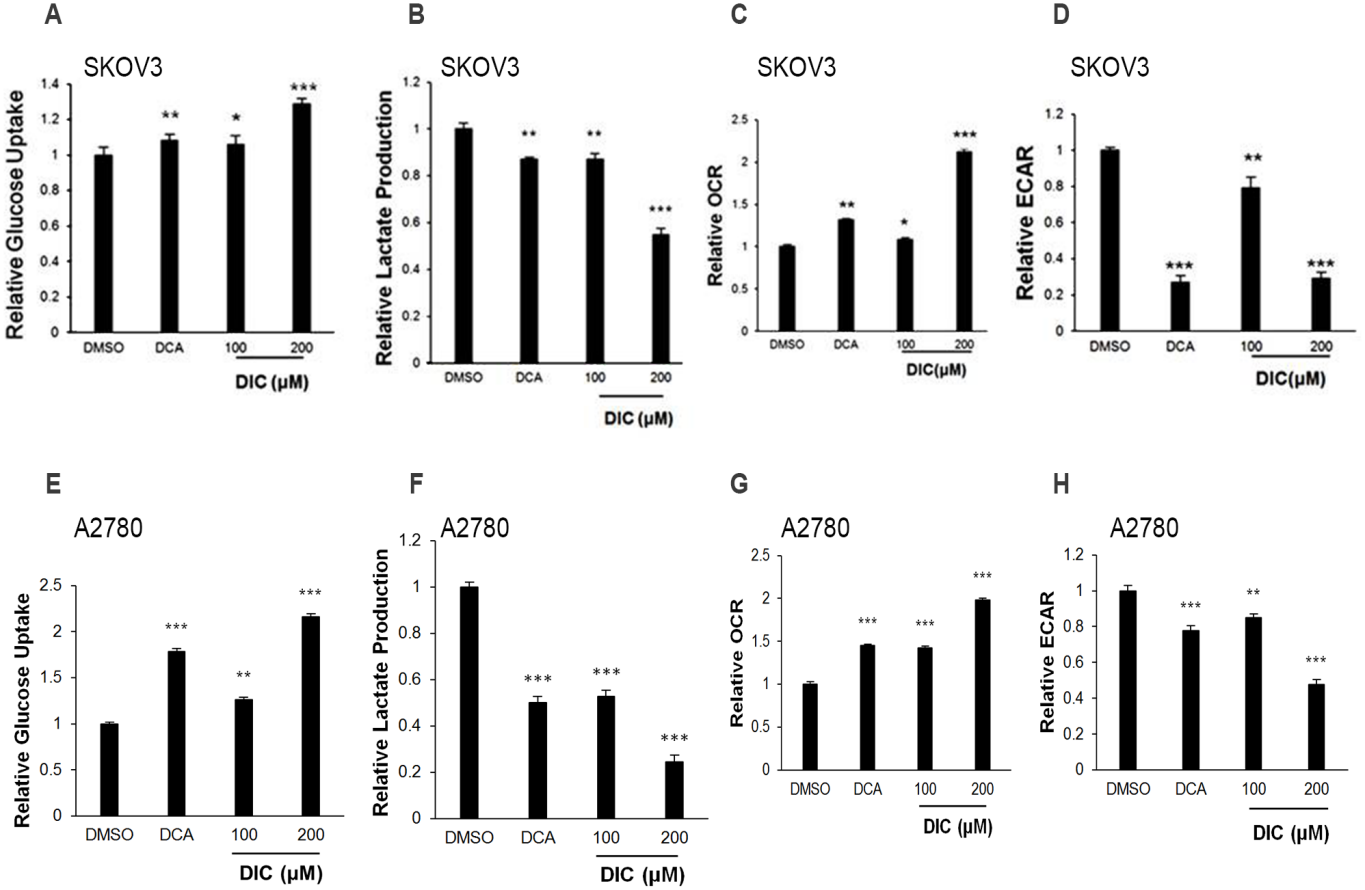
DIC switches glucose metabolism from aerobic glycolysis to oxidative phosphorylation in ovarian cancer cells. SKOV3 or A2780 cells were treated with vehicle (DMSO), DCA (50 mM), DIC (100 μM), or DIC (200 μM) for 24 h. Extracellular glucose **(A and E)** and lactate **(B and F)** concentrations were determined in the culture media and expressed as a normalization to total protein amounts. The oxygen consumption rate (OCR) **(C and G)** and extracellular acidification rate (ECAR) **(D and H)** were measured after the treatment of ovarian cancer cells. Data are presented as the mean ± SE from three independent experiments. *P < 0.05, **P < 0.01, ***P < 0.001, when compared to cells treated with DMSO.

PDK1, through the phosphorylation and inactivation of PDC, attenuates mitochondrial function and shifts glucose metabolism from mitochondrial oxidative phosphorylation to aerobic glycolysis. Considering that DIC potently inhibits the kinase activity of PDK1 both in a cell-free system and in ovarian cancer cells, we hypothesized that DIC would impact glucose metabolism and further the malignant behaviors of cancer cells. To test this hypothesis, we first measured the glucose uptake and the lactate production in culture media of DIC-treated SKOV3 cells. As shown in Fig 2A and 2B, glucose uptake was increased by 28.7% and lactate production decreased by 45.2% in response to treatment with 200 μM DIC. DCA and 100 μM DIC also significantly altered both parameters, but to a lesser extent (Fig 2A and 2B). Next, we quantified the OCR and ECAR upon DIC treatment in SKOV3 cells, the measure of the oxidative phosphorylation level and the glycolytic capacity, respectively. The results showed that DIC-treated SKOV3 cells exhibited a significantly higher OCR (212%) and a lower ECAR (29.1%) when compared to the vehicle (DMSO) group (Fig 2C and 2D).

Comparable phenotypes were also observed in A2780 cells, in which glucose uptake was increased by 116.2% and lactate production was reduced by 75.6% in response to the treatment with 200 μM DIC. DCA and 100 μM DIC also significantly altered both parameters, but to a lesser extent (Fig 2E and 2F). In addition, A2780 cells treated with 200 μM DIC exhibited a significantly higher OCR (198%) and a lower ECAR (47.6%), when compared to those treated with vehicle (DMSO) (Fig 2G and 2H).

### DIC enhances ROS production and attenuates the MMP level in ovarian cancer cells

**Fig 3:**
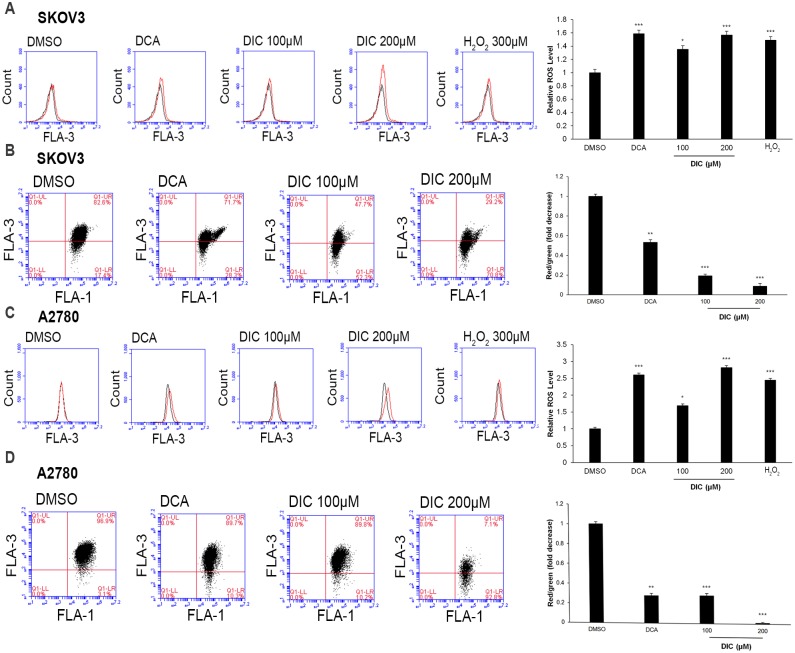
DIC elevates the reactive oxygen species (ROS) level and disrupts the mitochondrial membrane potential (MMP). Upon treating SKOV3 or A2780 cells with 2% DMSO, 50 mM DCA, 100 μM DIC, or 200 μM DIC for 24 h, the ROS generation **(A and C)** and MMP level **(B and D)** were measured by flow cytometry. *P < 0.05, **P < 0.01, ***P < 0.001, when compared to cells treated with DMSO.

To analyze the functional consequence of DIC-induced glucose metabolism in SKOV3 or A2780 cells, we first measured the mitochondrial ROS production and MMP levels. Consistent with the findings on glucose metabolism, treatment of SKOV3 cells with either DCA or DIC generated higher levels of ROS (149.0% in 300 μM H_2_O_2_-, 158.6% in DCA-, 135.3% in 100 μM DIC-, and 156.7% in 200 μM DIC-treated cells vs. 100% in DMSO-treated cells; Fig 3A) and a lower MMP (53.3% in DCA-, 19.2% in 100 μM DIC-, and 8.7% in 200 μM DIC-treated cells vs. 100% in DMSO-treated cells; Fig 3B). Similar alterations were also observed in A2780 cells in response to DIC treatment, where the ROS levels were elevated (244.6% in 300 μM H_2_O_2_-, 260.3% in DCA-, 169.0% in 100 μM DIC-, and 282.0% in 200 μM DIC-treated cells vs. 100% in DMSO-treated cells; Fig 3C), while the MMP levels were decreased (27.9% in DCA-, 28.2% in 100 μM DIC-, and 1.2% in 200 μM DIC-treated cells vs. 100% in DMSO-treated cells; Fig 3D). These data suggest that by targeting PDK1 and altering glucose metabolism, DIC robustly increases ROS production and impairs mitochondrial function.

### DIC inhibits cell viability and induces apoptosis in ovarian cancer cells

**Fig 4:**
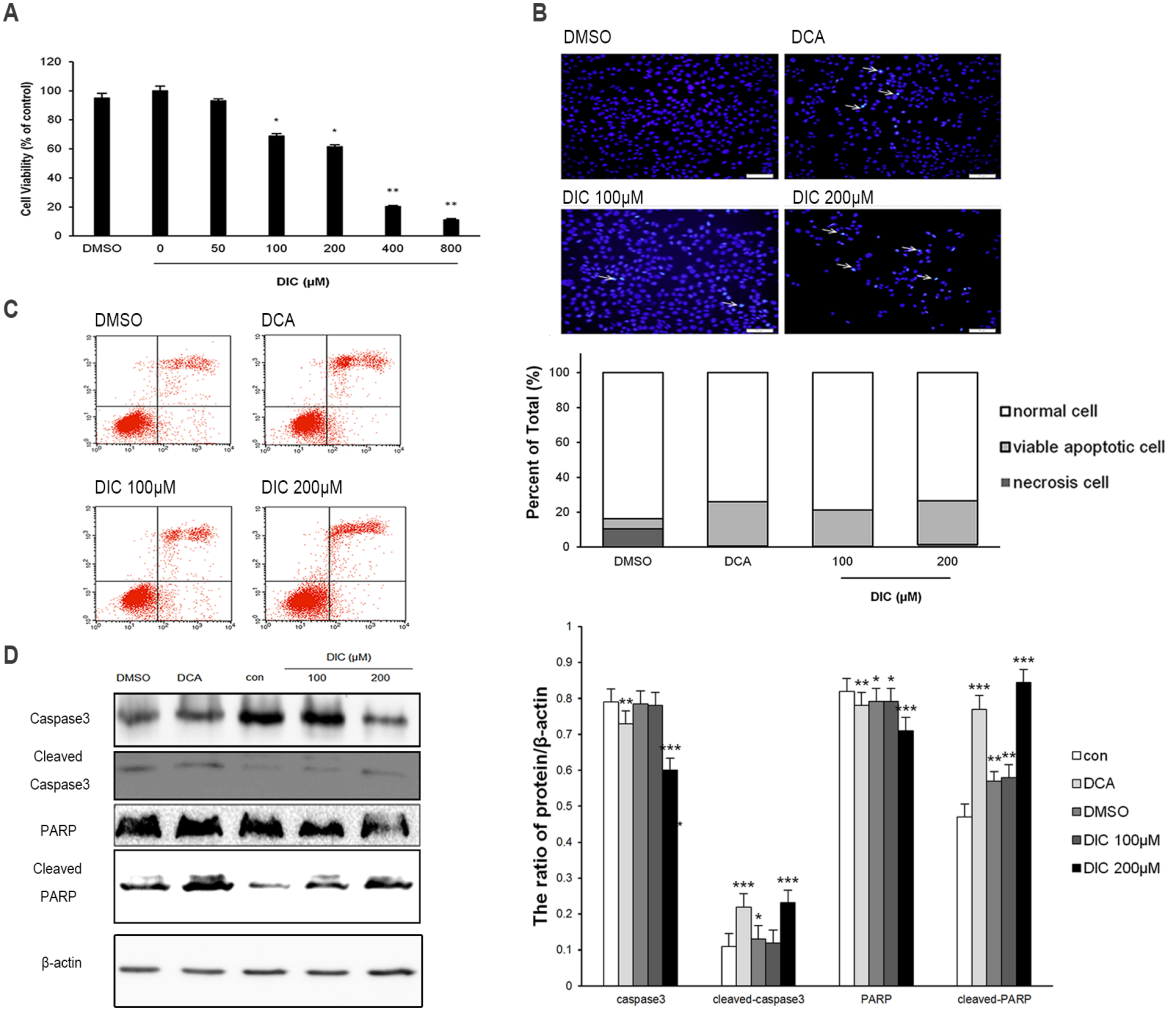
DIC inhibits cell viability and induces apoptosis in SKOV3 cells. **(A)** SKOV3 cells were treated with increasing concentrations of DIC for 24 h, and cell viability was measured by the MTT assay. **(B)** After the indicated treatments for 24 h, SKOV3 cells were stained with Hoechst 33342 and imaged by confocal microscopy (scale bar, 50 μm; arrows, Hoechst-positive apoptotic cells). **(C)** Cells were treated as indicated for 24 h, stained with annexin V and PI, and analyzed by flow cytometry. Representative flow cytometry images are shown on the left, and the quantification of annexin V^+^PI^+^ apoptotic cells is shown on the right. **(D)** Upon treatment, the protein levels of caspase-3, cleaved caspase-3, PARP, and cleaved PARP in SKOV3 cells were examined by western blot, with representative western blot images presented on the left and the quantitation of protein expression relative to β-actin (internal control) presented on the right. Data are presented as the mean ± SE from three independent experiments, *P < 0.05, **P < 0.01, ***P < 0.001, when compared to nontreated control cells.

Next, we examined the cell viability and apoptosis of DIC-treated SKOV3 or A2780 cells, two critical phenotypes for malignant tumor cells. The MTT assay showed that DIC inhibited the viability of SKOV3 cells in a dose-dependent manner within the concentration range of 50–500 μM; and the cytotoxicity induced by the vehicle (2% DMSO) was minimal and thus could be ignored (Fig 4A). To understand whether the reduced viability of SKOV3 cells in response to DIC treatment was due to any alterations in cell apoptosis, we treated SKOV3 cells with DMSO (2%), DCA (50 mM), DIC (100 μM), or DIC (200 μM) for 24 h and detected condensed chromatin (a sign of apoptosis) by Hoechst 33342 staining. As shown in Fig 4B, both 100 μM and 200 μM DIC markedly induced apoptosis of SKOV3 cells. Similarly, flow cytometric analysis of annexin V^+^PI^+^ cells revealed that 100 μM and 200 μM DIC treatment generated approximately 20.87% and 24.94% apoptotic cells, respectively, significantly higher than vehicle treatment (Fig 4C). Consistently, by examining the expression of the apoptotic effector caspase and its substrate (caspase-3, cleaved caspase-3, PARP, and cleaved PARP), we showed that in DIC-treated SKOV3 cells, the protein levels of cleaved caspase-3 and cleaved PARP increased, while those of caspase-3 and PARP decreased, compared with nontreated (control) or vehicle (DMSO)-treated cells (Fig 4D).

**Fig 5:**
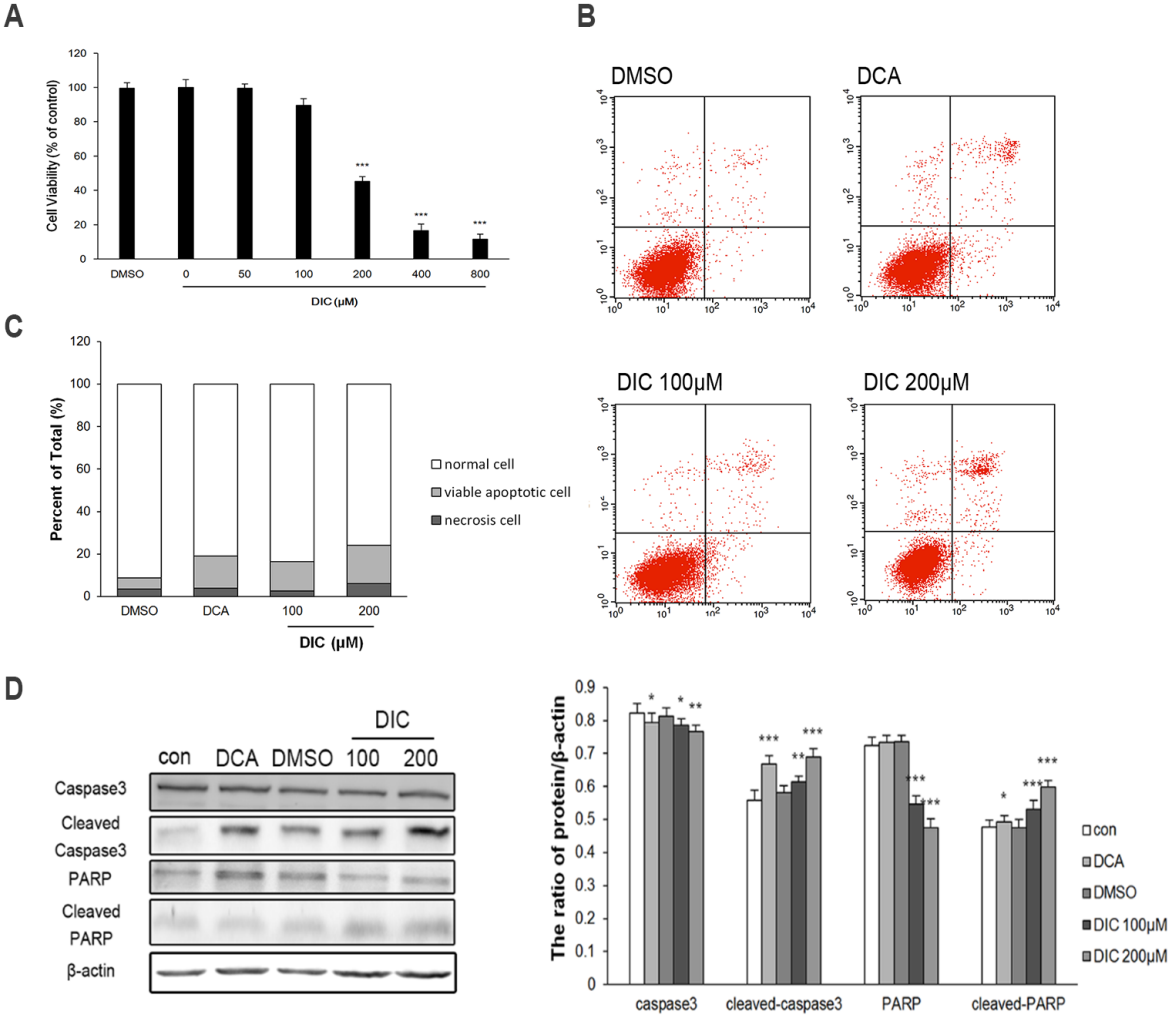
DIC inhibits cell viability and induces apoptosis in A2780 cells. **(A)** A2780 cells were treated with increasing concentrations of DIC for 24 h, and cell viability was measured by the MTT assay. **(B)** After the indicated treatments for 24 h, A2780 cells were stained with annexin V and PI, and analyzed by flow cytometry. **(C)** The quantification of annexin V^+^PI^+^ apoptotic cells. **(D)** Upon treatment, the protein levels of caspase-3, cleaved caspase-3, PARP, and cleaved PARP in A2780 cells were examined by western blot, with representative western blot images presented on the left and the quantitation of protein expression relative to β-actin (internal control) presented on the right. Data are presented as the mean ± SE from three independent experiments, *P < 0.05, **P < 0.01, ***P < 0.001, when compared to nontreated control cells.

In A2780 cells, we also observed dose-dependent reduction of cell viability in response to DIC (Fig 5A). DIC at 100 μM or 200 μM generated approximately 16.56% and 24.18% annexin V^+^PI^+^ apoptotic cells, respectively, significantly higher than the vehicle treatment (Fig 5B and 5C). Concomitant with the reduced p-PDHA1 level, cleaved caspase and its substrate cleaved PARP were increased in DIC-treated A2780 cells, as in DIC-treated SKOV3 cells (Figs 1D and 5D).

### DIC inhibits tumor growth and induces cell apoptosis in SKOV3 xenografts

**Fig 6:**
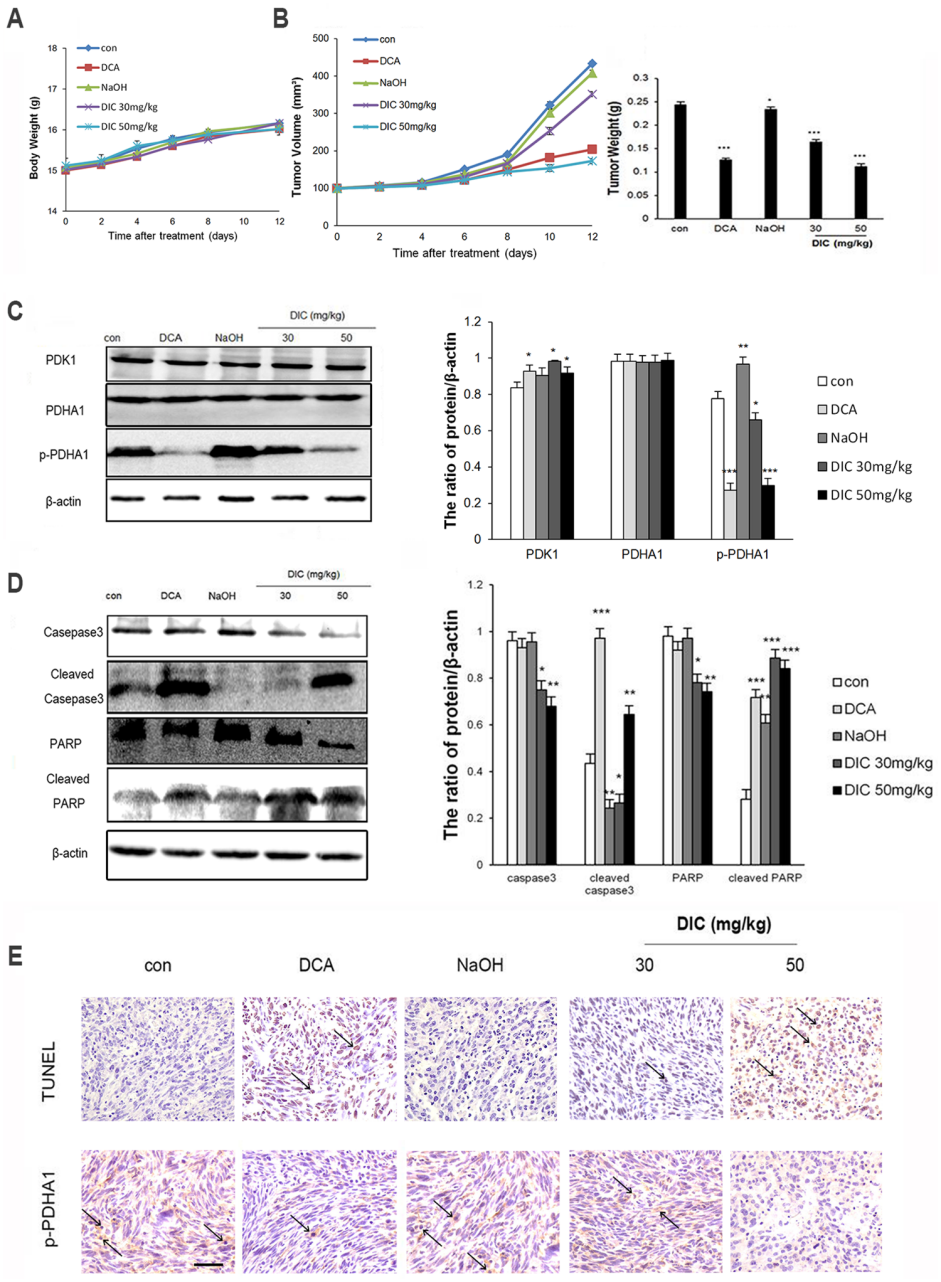
DIC suppresses tumor growth *in vivo*. SKOV3 xenografts were established in nude mice, and the mice were treated as indicated. **(A)** The body weights of mice from all groups were monitored during the 12-day treatment period. **(B)** The tumor volume and tumor weight were measured at the indicated time points during treatment and at the time of sacrifice, respectively. **(C)** The protein levels of PDK1, PDHA1, and p-PDHA1 were examined by western immunoblotting, with β-actin examined as the internal control. Representative western blot images are shown on the left, and the quantification of protein levels relative to β-actin are shown on the right. **(D)** The protein levels of caspase-3, cleaved caspase-3, PARP, and cleaved-PARP in the SKOV3 xenografts were examined by western immunoblotting. Representative western blot images are shown on the left, and the quantification of protein levels relative to β-actin are shown on the right. Data are presented as the mean ± SE for all mice in each group. *P < 0.05, **P < 0.01, ***P < 0.001, when compared to the control group. **(E)** Detection of apoptosis and p-PDHA1 by the TUNEL assay and immunohistochemistry, respectively, in xenograft samples from the indicated groups (scale bar, 100 μm; arrows, apoptotic cells).

To further characterize the anticancer activity of DIC, we established SKOV3 xenografts in nude mice and treated them with normal saline (control), vehicle (1 mM NaOH), DCA (100 mg/kg), DIC (30 mg/kg), or DIC (50 mg/kg). During the 12-day treatment, the body weight from all groups of mice did not significantly change, suggesting that all treatments were safe for the animals (Fig 6A). However, DCA at 100 mg/kg, DIC at 30 mg/kg, and DIC at 50 mg/kg all significantly reduced tumor volume and decreased tumor weight, when compared to tumors from control or vehicle groups (Fig 6B).

To understand the molecular mechanisms underlying DIC-induced tumor inhibition, we first measured the protein levels of PDK1, PDHA1, and p-PDHA1 in xenografts by western blot. Consistent with our findings *in vitro*, although all treatments did not alter the PDK1 level, DCA and DIC (at two different doses) significantly reduced the level of p-PDHA1, suggesting that DIC significantly inhibited the enzymatic activity of PDK1 *in vivo* (Fig 6C). Next, we examined the expression of the apoptotic markers caspase-3, cleaved caspase-3, PARP, and cleaved-PARP in SKOV3 xenografts (Fig 6D). We showed that cleaved caspase-3 and cleaved PARP were significantly increased, while total caspase-3 and total PARP were significantly decreased in DIC-treated SKOV3 xenografts, when compared to tumors from the control or vehicle group. Lastly, we detected apoptosis in SKOV3 xenografts using a TUNEL assay and examined p-PDHA1 by immunohistochemistry. We observed significantly higher TUNEL^+^ signals, which correlated with a reduced p-PDHA1 level in DIC-treated tumor samples, when compared to samples from the control or vehicle group (Fig 6E). These results suggest that DIC, by targeting the kinase activity of PDK1, induces apoptosis of tumor cells *in vivo*.

## Discussion

In this study, we showed for the first time that DIC potently inhibits the kinase activity of PDK1 in a cell-free biochemical reaction system, in cultured SKOV3 and A2780 cancer cells *in vitro*, and in SKOV3 xenografts *in vivo*. Correlating with PDK1 inhibition, DIC switches glucose metabolism in SKOV3 and A2780 cells from aerobic glycolysis to oxidative phosphorylation, elevates ROS production, reduces the MMP level, stimulates apoptosis, reduces the viability of these cells, and suppresses xenograft growth.

As an essential molecule governing aerobic glycolysis that is upregulated in multiple human cancers, PDK1 has become a highly promising target for anticancer therapy [44–46]. So far, few inhibitors of PDK1 have been in clinical trials for cancer treatment, potentially because only a limited number of PDK1 inhibitors are available. To identify novel and potent PDK1 inhibitors with minimal side effects, we have applied a computational approach to screen for potential PDK inhibitors [39]. The computer-aided molecular docking suggests that DIC, a coumarin compound, may disrupt the interaction between PDK1 and PDC through blocking the lipoamide-binding pocket of PDK1 (Fig 1A). This clue led to this study, where through the in-well kinase assay as well as the measurement of PDHA1 and p-PDHA1 protein levels both in SKOV3 and A2780 cells and in SKOV3 xenografts, we showed that DIC potently inhibits PDK1 activity both *in vitro* and *in vivo*.

Discovering new pharmacological activities for old drugs offers the advantage that the pharmacokinetic and toxicological profiles (a major goal for phase I and II clinical trials) are, most of the time, well characterized. DIC has been used as an anticoagulant in the clinic for more than 60 years and has shown minimal side effects and a high safety profile [47]. Therefore, further trials on DIC in cancer therapy, if any, would experience minimal optimization and thus a significantly reduced cost for regimen and safety profiling. In addition, DIC presents a superior binding affinity to PDK1. The molecular complex binding affinity from the CDOCKER interaction energy was determined to be -33.65 kcal/mol. The in-well kinase assay showed that the IC_50_ for DIC was 19.42 ± 0.032 μM, more than 150-fold lower than that for DCA (3.042 ± 0.087 mM), the most advanced PDK1 inhibitor in clinical trials for cancer therapy [48]. Consistently, in our *in vitro* studies, DIC at 100 or 200 nM achieved comparable, if not better, results on multiple phenotypes than 50 mM DCA. For the *in vivo* studies, we also took into consideration the safety profiles of DIC. We administered DIC intraperitoneally at a maximal dose of 50 mg/kg every other day, which equals to 162 mg/d in humans and is below the maximal anticoagulant dose currently used (350 mg/d in the USA [49] and 300 mg/d in China [50]). By monitoring the body weight of mice during the treatment, we confirmed that the doses we administered are safe. Although the subcutaneous xenograft is not an ideal model for ovarian cancer, the proof-of-principle evidence obtained from it supports the antitumor activities of DIC and justifies further preclinical and clinical studies on DIC in ovarian cancer.

The activities of PDK1 on glucose metabolism are carried out via binding to PDC, phosphorylating PDHA1, and inactivating PDC. Functionally, PDK1 enhances glucose consumption, attenuates lactate production, increases oxygen consumption, and reduces extracellular acidification [16, 51–53]. Consistently, our results showed that DIC elevates the glucose uptake and OCR, while decreasing the lactate production and ECAR in SKOV3 and A2780 cells (Fig 2). These results indicate that inhibiting PDK1 by DIC shifts glucose metabolism from aerobic glycolysis to oxidative phosphorylation in ovarian cells.

Subsequent to the alteration in glucose metabolism, DIC treatment increases the ROS level and decreases the MMP in both SKOV3 and A2780 cells (Fig 3). PDK1 inhibition leads to an elevated influx of acetyl-CoA into the Krebs cycle, promotes NADH delivery to complex I of the electron transport chain, and induces an elevated production of superoxide or ROS, which in turn damages redox-sensitive complex I and the proton pump. Protons cannot efflux through the mitochondrial inner membrane, resulting in a decreased MMP [18, 54, 55]. Cancer cells exhibit a hyperpolarized MMP compared with normal cells [53]. Depolarizing the MMP opens the mitochondrial transition pore, giving rise to the efflux of cytochrome c that causes the cleavage of the pro-apoptotic protein caspase-3 and PARP and eventually activating the caspase-independent signaling pathway of apoptosis [54, 56]. Consistently, we found that the levels of cleaved caspase-3 and cleaved PARP increased, which are associated with elevated apoptosis, reduced viability, and suppressed tumor growth, as detected by multiple assays both *in vitro* and *in vivo*.

Several studies have examined the relationship between PDHA1 expression and the prognosis and survival of ovarian cancer patients. Li *et al*. have reported that a reduced PDHA1 level led to an unfavorable prognosis in ovarian cancer patients and observed PDHA1 reduction in three different ovarian cancer cell lines including SKOV3 cells [57]. Zhao *et al*. have discovered that overexpressing PDH protein could abolish the oncogenic effects of miR-203, including promoting the metastasis of ovarian cancer cells *in vivo* and shortening the survival of the tumor-bearing nude mice [58]. Taken together, these studies suggest that a high PDH level correlates with a favorable prognosis and a prolonged survival of ovarian cancer patients.

In summary, this is the first study to show that DIC is a potent inhibitor of PDK1. By inhibiting the kinase activity of PDK1, DIC initiates a cascade of alterations in multiple phenotypes of ovarian cancer SKOV3 and A2780 cells, including antagonizing the Warburg effect, increasing ROS production, and inducing apoptosis, which leads to effective inhibition of tumor growth *in vivo*. In addition, we identified a new inhibitor of PDK1, revealed that DIC has novel pharmacological activity, and most importantly provided preclinical evidence that DIC may be a safe and effective anticancer drug.
